# Unlocking the mystery of the mid-Cretaceous Mysteriomorphidae (Coleoptera: Elateroidea) and modalities in transiting from gymnosperms to angiosperms

**DOI:** 10.1038/s41598-020-73724-7

**Published:** 2020-10-08

**Authors:** David Peris, Robin Kundrata, Xavier Delclòs, Bastian Mähler, Michael A. Ivie, Jes Rust, Conrad C. Labandeira

**Affiliations:** 1 Institute of Geosciences, University of Bonn, 53115 Bonn, Germany; 2 Department of Zoology, Faculty of Science, Palacky University, 77900 Olomouc, Czech Republic; 3 Departament de Dinàmica de la Terra i de l’Oceà and Institut de Recerca de la Biodiversitat (IRBio), Facultat de Geologia, Universitat de Barcelona, 08028 Barcelona, Spain; 4 Montana Entomology Collection, Montana State University, Bozeman, MT 59717 USA; 5 Department of Paleobiology, National Museum of Natural History, Smithsonian Institution, Washington, DC 20013 USA; 6 Department of Entomology and Behavior, Ecology, Evolution and Systematics Program, University of Maryland, College Park, MD 20742 USA; 7 College of Life Sciences, Capital Normal University, Beijing, 100048 China

**Keywords:** Evolution, Palaeontology, Taxonomy

## Abstract

The monospecific family Mysteriomorphidae was recently described based on two fossil specimens from the Late Cretaceous Kachin amber of northern Myanmar. The family was placed in Elateriformia *incertae sedis* without a clear list of characters that define it either in Elateroidea or in Byrrhoidea. We report here four additional adult specimens of the same lineage, one of which was described using a successful reconstruction from a CT-scan analysis to better observe some characters. The new specimens enabled us to considerably improve the diagnosis of Mysteriomorphidae. The family is definitively placed in Elateroidea, and we hypothesize its close relationship with Elateridae. Similarly, there are other fossil families of beetles that are exclusively described from Cretaceous ambers. These lineages may have been evolutionarily replaced by the ecological revolution launched by angiosperms that introduced new co-associations with taxa. These data indicate a macroevolutionary pattern of replacement that could be extended to other insect groups.

## Introduction

Recently, there have been instances of fossil beetles described from the mid Cretaceous that have included several studies illustrating the role beetles have played in the major transformation of the continental biota^1–6^. This field has expanded rapidly mostly because of Cretaceous amber studies that document a recent increase in the number of new fossil taxa of Coleoptera and, equally important, highly relevant paleoecological studies resulting in major macroevolutionary patterns^7,8^.

Currently, Coleoptera consist of approximately 400,000 described species and represent arguably the largest and most diverse radiation of animals on the planet. However, the study of fossil beetles is not limited solely to documenting the diversity of life, but also explains how and why this group has come to so dominate terrestrial biodiversity^9^. One such pattern, occurring at the midpoint of beetle deep-time history, is the relationship of beetles with gymnosperms and angiosperms during the later Early Cretaceous from about 125 to 100 million years ago.

Among the slightly more than 80 beetle families with at least one representative described from Cretaceous ambers^8^, six are solely fossil lineages: Elodophthalmidae and Tetrameropseidae from Lebanese amber and Apotomouridae, Mesophyletidae, Mysteriomorphidae, and Passalopalpidae from Kachin amber (see references and details in^8^). These lineages represent a relatively high proportion of taxa for the low extinction rate over the long evolutionary history of Coleoptera^9–11^. Such fossil lineages also contribute to increasing the extinction rates for insects in general^12–14^. The evolutionary turnover of beetles associated with ecological diversification of angiosperms during the later Early Cretaceous has been attributed to structural adaptations by specialized herbivorous beetles that were engendered by their co-associations with flowering plants^10,11,15,16^. However, this hypothesis has been questioned^2,17^. Accordingly, the rise of angiosperms during the Cretaceous may not have caused an increase in family-level diversity in insects^12,14,18^, not in Coleoptera^2,17^, and moreover may have caused a subtle decline in diversity into the Late Cretaceous^13,19^. Nevertheless, molecular phylogenetic studies focusing on specific groups indicate that at least some of these taxa appear to have diversified extensively during the Cretaceous in response to newly formed niches^20^. The generally cited decrease in diversity, however, may have been responsible for a relatively high number of beetle families described in the amber record from the Cretaceous that currently do not exist.

One of these fossil families, Mysteriomorphidae, recently was established based on two specimens described as *Mysteriomorphus pelevini* Alekseev and Ellenberger, 2019. These specimens were found in different amber samples from the earliest Late Cretaceous Kachin amber of northern Myanmar. The new taxon was originally classified as Elateriformia *incertae sedis*, and the genus and family name were designated based on a unique combination of characters that are present variously in different lineages of Elateroidea and Byrrhoidea^21^. The phylogenetic relationships within Elateriformia, as well as within Elateroidea and Byrrhoidea, are far from fully understood, despite the effort of numerous recent morphological and molecular-based studies^22–26^. Moreover, the morphology of taxa within this lineage should be treated carefully when establishing a classification, attributable to the independent evolutionary origins of the soft-body condition, neoteny and other confounding traits within the encompassing clade^24–27^.

In this study, the detailed examination of four additional specimens of Mysteriomorphidae from the same deposit as the type series, together with the virtual reconstruction of one of these specimens using a CT-scan analysis, facilitated a more objective diagnosis of this lineage. Based on re-evaluation of all available morphological characters, we definitively place Mysteriomorphidae in Elateroidea. Moreover, we discuss a possible reason for the family-level stability observed in insect lineages during the Cretaceous using the example of beetles under the established transitional modalities from gymnosperm to angiosperm hosts.

## Systematic paleontology

**Figure 1:**
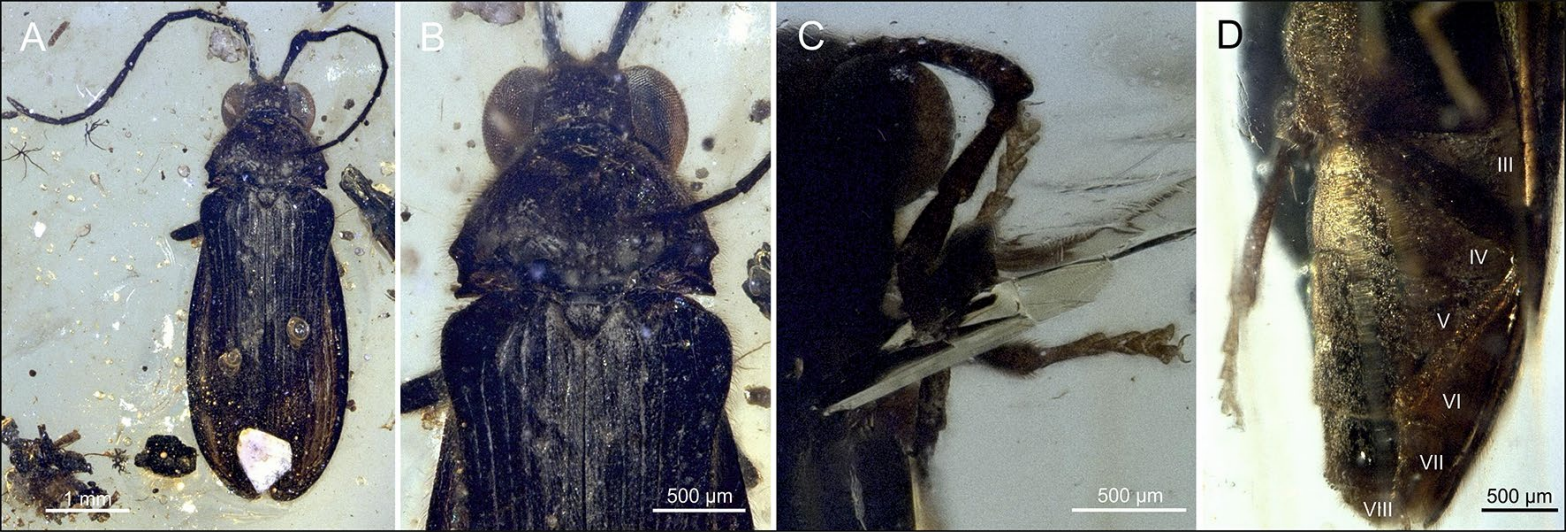
Morphology of *Mysteriomorphus pelevini* Alekseev and Ellenberger, 2019. (**A**) Dorsal view of specimen NIGP173649. (**B**) Detail of the pronotum and proximal part of the elytra of specimen NIGP173649. (**C**) Protarsi of specimen NIGP173651. (**D**) Abdomen of specimen NIGP173651; sternites III–VIII are marked.

**Figure 2:**
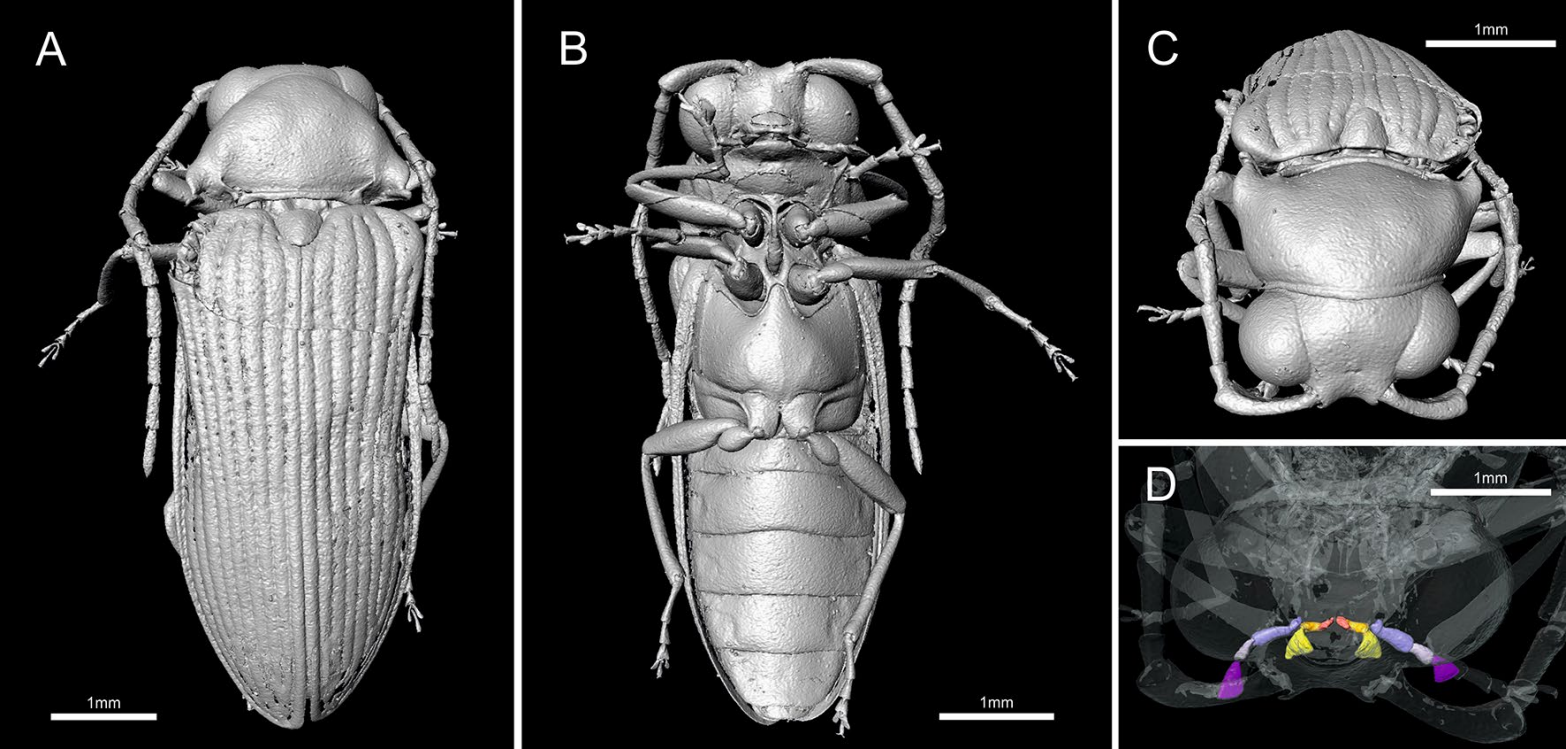
Micro-CT reconstruction of *Mysteriomorphus pelevini* Alekseev and Ellenberger, 2019, specimen NIGP173651. (**A**) Dorsal habitus. (**B**) Ventral habitus. (**C**) Oblique view of the head and pronotum. (**D**) Oblique view of the maxillary and labial palpi.

- Order Coleoptera Linnaeus, 1758.
- Suborder Polyphaga Emery, 1886.
- Series Elateriformia Crowson, 1960.
- Superfamily Elateroidea Leach, 1815.
- Family Mysteriomorphidae Alekseev and Ellenberger, 2019
- Type genus: *Mysteriomorphus* Alekseev and Ellenberger, 2019
- Figures 1–2.

### Systematic placement

Mysteriomorphidae are herein transferred from Elateriformia *incertae sedis* to the superfamily Elateroidea, near Elateridae, based on a combination of diagnostic characters including the structure of prothorax and the connation of the first four abdominal ventrites (see below). The family rank of Mysteriomorphidae has been maintained due to their divergent morphology (see “Discussion” for more details).

### New diagnosis

Mysteriomorphidae are characterized by the following unique combination of characters: antennal insertions moderately separated, located next to the inner margin of the eye, the frons raised on the mesad side; antennae with 11 antennomeres, filiform to slightly serrate, with the scape being by far the longest antennomere; eyes conspicuously large, with their widest diameter measured at the level of the antennal insertions slightly less than the interocular distance; maxillary palpi long, with apical palpomere securiform; pronotum margined laterally, variably emarginate before posterior angles, base of pronotum with deep submarginal groove between a pair of deep pits, sublateral lines of variable length extending anteriorly on each side of disk from these pits, posterior angles of pronotum small, acute, produced laterally; prosternum transverse, with weakly developed chin-piece and a narrow prosternal process fitting into mesoventral cavity; pro- and mesocoxal cavities open; pro- and mesocoxae conical, projecting, both narrowly separated; elytra covering whole abdomen, striate, each elytron independently rounded apically; epipleura incomplete, gradually narrowed, not reaching the elytral apex; metacoxa with plate very weakly present only over trochanteral insertion and immediately adjacent margin of the conical portion of the coxa; tibial spurs double, all subequal in length; tarsi 5–5–5, with tarsomere IV deeply bilobed; claws simple; abdomen with six ventrites, the first four of which are connate.

### Redescription

Body length 2.5–7.3 mm (GPIH 4947 smallest, NIGP173648 largest); body (Figs. 1A, 2A) brown to black, elongate, subparallel-sided, pronotum, elytra, venter and legs covered with short, sparse, decumbent setae, laterally fringed with long erect subparallel setae (Fig. 1B).

Head hypognathous, subquadrate, visible from above, partially inserted into pronotum but not covering eyes, slightly declined, including eyes wider than anterior pronotal margin but narrower than pronotum at widest place. Eyes lateral, large, entire, strongly protuberant, hemispherical, finely faceted, without interfacetal setae (Fig. 1B); separated by slightly more than eye width. Frontoclypeal suture absent. Antenna inserted adjacent to inner margin of eye, separated by more than half of eye width; insertions exposed from above, mesially closed by raised portion of frons (Fig. 2C). Antenna long, usually slightly surpassing middle of elytra, with 11 antennomeres, filiform to slightly serrate. Scape very elongate, gradually slightly widened toward apex, distinctly longer than other antennomeres, three to four times as long as pedicel, less than twice as long as antennomeres III–X; pedicel attached subapically, longer than wide, shortest; antennomeres III to X elongate, about twice to three times as long as wide; antennomere XI slightly longer, apically subacuminate (Figs. 1A, 2B). Labrum visible, small, transverse, slightly rounded anteriorly. Mandible slender, unidentate, widened basally, gradually narrowed toward apex, moderately curved. Maxillary palpus long, reaching procoxa, with four palpomeres; basal palpomere very small, about as long as wide, much shorter than palpomere II; palpomere II elongate, about 1.4–1.7 times as long as palpomere III; palpomere III longer than wide; apical palpomere longest, about twice as long as palpomere III, securiform, apically expanded and flattened. Labial palpus short, with three palpomeres; basal palpomere distinctly shortest, about long as wide; palpomere II elongate, slender; apical palpomere longer than palpomere II, securiform, apically expanded and flattened (Fig. 2D).

Pronotum transverse, about 1.8 times wider than long, widest subposteriorly, gradually narrowing anteriorly; anterior margin rounded; anterior pronotal angles inconspicuous, broadly rounded; sides narrowly explanate, margined, forming lateral carina, abruptly emarginate before posterior angles, with that emargination simple (Fig. 2A) or with additional inner emargination (Fig. 1B); posterior margin (Fig. 2A) weakly bisinuate, carinate; posterior angles short, narrow, acute, produced posterolaterally (Figs. 1B,C, 2A); base of pronotum with deep submarginal groove between a pair of deep pits; with sublateral longitudinal carinae running from posterior pits on either side of pronotal disc, either reaching about middle (Fig. 1A,B) or basal third of disk (Fig. 2A,C); pronotal disk convex. Prosternum (excluding prosternal process) wider than long, slightly longer than one procoxal diameter, chin-piece short, rounded and slightly bent downwards; prosternal process about as long as prosternum itself, narrow, subparallel-sided basally, slightly widened subapically, rounded apically, gradually slightly curved upwards from lateral view, with apex fitting into mesoventral cavity (Fig. 2B). Procoxal cavities open, narrowly separated by prosternal process; protrochantin hidden. Scutellar shield about as long as wide (slightly wider than long in holotype and smaller specimens, slightly longer than wide in larger specimens), anterior margin more-or-less straight, posterior margin simply rounded. Mesoventrite shorter than mesocoxal diameter, with mesoventral process reaching about middle of mesocoxal cavity; mesoventral cavity relatively small but well-defined; mesocoxal cavities open, narrowly separated. Metaventrite slightly wider than long, slightly convex, discrimen long; metaventral process reaching middle of mesocoxal cavities, apically rounded. Metepisternum narrow, subparallel-sided, slightly widened posteriorly. Elytra oblong-elongate, fully covering abdomen, 1.9–2.2 times longer than combined width of both elytra; widest just after middle, slightly wider than pronotum, dorsally moderately convex, with well-developed humeri; margins explanate; apices separately rounded; pubescence short and fine as in pronotum (Fig. 1B). Each elytron with nine well-developed striae (including lateral stria), lacking scutellary striole; punctures in striae moderately large; interstriae convex; pubescence relatively short, with setae about as long as distance between striae, semi-erect, directed posteriad (Fig. 2A). Epipleuron incomplete, widest in humeral area, gradually narrowed, not reaching elytral apex (Fig. 2B). Hind wings present. Legs long, slender, pro- and mesocoxae separated, conical; metacoxae contiguous, transverse, strongly conical internally, and extending laterally to meet elytral epipleura; metacoxa with posterior face, metacoxal carina distinct, plate very weakly present only over trochanteral insertion and immediately adjacent margin of the conical portion of the coxa. Femur and tibia elongate; femur shorter than tibia, grooved to receive tibia; all tibiae with paired apical spurs, although often indistinct. Tarsi pentamerous; tarsomere I longest, approximately twice as long as tarsomere II in meso- and metalegs, less than twice in forelegs; tarsomere II widened apically; tarsomere III shortest, widened and emarginate apically; tarsomere IV deeply bilobed; apical tarsomere elongate, slender, narrow; claws slender, moderately long, symmetrical, falcate, acute.

Abdomen with six ventrites (sternites III–VIII), the first four of which are connate (Fig. 1D). Ventrite 1 slightly shorter than ventrite 2, with intercoxal process not visible; ventrites 2–4 of subequal length; ventrite 5 apically more or less straight; ventrite 6 shorter than previous ventrites, widely rounded apically.

### Family composition

One genus with a single described species.

Genus *Mysteriomorphus* Alekseev and Ellenberger, 2019.

### Type species

*Mysteriomorphus pelevini* Alekseev and Ellenberger, 2019, by original designation (Figs. 1–2).

### Type locality

Northern Myanmar, Kachin State, near the town of Tanai (Danai); Late Cretaceous (early Cenomanian) in age^28^.

### Type material

GPIH 4947 (Holotype) and GPIH 4948 (Paratype), both specimens deposited in the amber collection of the Center for Natural History (CenNak), University of Hamburg, Germany.

### Morphological variability

Alekseev and Ellenberger^21^ reported differences between the holotype and paratype in the body length and shape, and the shapes of the antennae and pronotum. They attributed those differences to potential sexual dimorphism (although the sex of the type specimens was unknown) and to the paratype deformation caused by preservation processes, and they considered both specimens a single species. We observed similar differences in body size and morphology among the specimens examined here. The larger specimens (NIGP173648 and NIGP173651) have a more convex body, scutellar shield slightly longer than wide, pronotum with a simple emargination before posterior angles, and with sublateral longitudinal carinae reaching only about the basal third (Fig. 2A–C). Alternatively, smaller specimens (NIGP173649 and NIGP173650) have a generally more flattened body, scutellar shield slightly wider than long, pronotum with a double emargination before posterior angles, and with sublateral longitudinal carinae reaching to about half of the pronotal length (Fig. 1A, B). However, these characters are not consistent when we take into consideration also the type material of *M. pelevini*. The holotype is a small specimen but with pronotum as in NIGP173648 and NIGP173651, and the large paratype resembling the pronotum shape of the newly examined small specimens NIGP173649 and NIGP173650 (Figs. 1 and 2^21^). Additionally, we do not know the sex of any of the specimens. Some variation, such as the pronotal sublateral carinae or shape of the scutellar shield are not clearly visible in some specimens, and the impact of deformation is unknown in all the observed variation in shape. We strongly suspect that multiple species are present in the material examined, but we tentatively treat all specimens as conspecific until more material is available for a more detailed morphological examination.

## Discussion

### Systematic position of mysteriomorphidae

In the original description, Mysteriomorphidae were placed as Elateriformia *incertae sedis*, and the authors discussed their affinities either to Byrrhoidea or to Elateroidea^21^. Regarding Byrrhoidea, only Ptilodactylidae were taken into consideration as potentially related to Mysteriomorphidae by the authors. However, the authors did not explicitly provide any evidence for Ptilodactylidae as closely related to Mysteriomorphidae, other that they are “similar-looking”. We found no support for a relationship between Mysteriomorphidae and Ptilodactylidae.

After study of the new material, we found evidence for a relationship with Elateroidea, and specifically with Elateridae. We re-examined the type material of Mysteriomorphidae from high-resolution photographs and studied in detail additional specimens using CT-scan analysis, providing a highly resolved reconstruction of the body (Fig. 2). Based on our results, we supplemented and improved the original diagnosis of Mysteriomorphidae, and corrected several morphological characters which were misinterpreted by, or not available to, Alekseev and Ellenberger^21^. These include the absence of a frontoclypeal suture (vs. presence in the original description), antennal insertions located adjacent to the inner margin of the eyes, separated by more than half the width of an eye (vs. almost contiguous and located frontally), posterior angles of the pronotum produced posterolaterally (vs. posteriorly), the prosternal process elongate, about as long as the prosternum in front of the procoxae (vs. indistinct, not visible between procoxae), the procoxae narrowly separated by a prosternal process (vs. contiguous), the metacoxae contiguous (vs. narrowly separated), the tibial apical spurs paired (vs. one), and the abdomen with six ventrites, the first four of which are connate (vs. five, without any indication how many are connate). The combination of diagnostic characters, including the pro- and mesothoracic, and abdominal structures, clearly indicates that the Mysteriomorphidae belong within the superfamily Elateroidea.

Elateroidea currently include families which were earlier classified in the former Artematopoidea, Elateroidea sensu stricto (i.e., well-sclerotized groups with a pro-mesothoracic clicking mechanism), and Cantharoidea (i.e., soft-bodied groups)^24,29^. Recent molecular phylogenetic analyses show that none of the previously defined superfamilies are monophyletic, and both well-sclerotized and soft-bodied lineages are present in the early-branching groups (Artematopodidae, Omethidae, Cerophytidae, Jurasaidae, Eucnemidae, Throscidae and Brachypsectridae) as well as in the so-called "higher Elateroidea" (Lycidae, Iberobaeniidae, Lampyridae, Cantharidae, Phengodidae, Rhagophthalmidae, and Elateridae including Drilini, Cebrionini, Omalisinae, and Plastocerinae)^10,24,26,30–32^. Apparently, the soft-bodied condition originated multiple times within Elateroidea^24^, and the transitions from a completely to a poorly sclerotized body include various changes in external morphology, such as the loss of interlocking devices, reduction of the prosternum and mesoventrite, and expression of basal abdominal sternites as ventrites (for a review, see^27^). The independent origins, together with the different degrees of these traits in various elateroid lineages, cause problems in phylogenetic analyses based on morphology alone^22^.

For fossil Mysteriomorphidae, somewhat incomplete morphological data are the only source of information we have, making identification of their putative relatives rather challenging. The elongate prosternal process, which fits into the mesoventral cavity, together with four connate abdominal ventrites (Fig. 1D), exclude all soft-bodied families (e.g., Lycidae, Lampyridae, Cantharidae). The only elateroid fossil family, Berendtimiridae, shares with Mysteriomorphidae the distinctly elongate scape, the bilobed tarsomere IV, and six abdominal ventrites, but it clearly differs in a presumed soft body, a differently formed head without antennal sockets, antennomere II only slightly shorter than antennomere III, and a pronotum that is widest posteriorly, without distinct emarginations near the posterior angles^33^. Brachypsectridae have different structure of the head, antennae either with 11 or 12 antennomeres, some of which either form a pectinate club or are bipectinate, and the abdomen with five free ventrites^34^. Artematopodidae, Throscidae and Eucnemidae (except for a few derived genera) differ from Mysteriomorphidae in having a much wider prosternal process and five connate, abdominal ventrites. Additionally, Artematopodidae have a different head form, with the antennal insertions widely separated and not raised, and a unique elytral locking system not found in Mysteriomorphidae. Throscidae and Eucnemidae (except for *Anischia* Fleutiaux) have well-developed metacoxal plates^22^. Cerophytidae are excluded by their unique, flat hind coxae and the shape of the prosternal process that has a pair of subapical lateral projections forming secondary procoxal articulations^35^.

The most morphologically similar of the elateroid families to Mysteriomorphidae are Elateridae. As currently defined, Elateridae include not only the typical well-sclerotized click-beetles, but also several lineages with variously modified morphology due to neotenic development^24,32^. Consequently, such a widely delimited group as Elateridae is difficult to diagnose morphologically. The characters used to diagnose Mysteriomorphidae can be found individually in some Elateridae. Therefore, Mysteriomorphidae could represent a morphologically derived lineage within Elateridae, possibly weakly affected by neoteny. However, since Mysteriomorphidae are unique in the specifics of the “non-elaterid” head, including the frontoclypeal region, and have a unique combination of characters that include the very long scape, distinctly emarginate area of the pronotum lateral margin before the posterior angles, and the deeply bilobed tarsomere IV, we retain here the current taxonomic status and provisionally consider them a distinct family. Additional, well-preserved specimens of both sexes with clearly visible ventral parts and at least partly exposed genitalia could provide further diagnostic characters and assist us to better evaluate the systematic position of Mysteriomorphidae.

### Evolution of beetle families during Cretaceous

**Figure 3:**
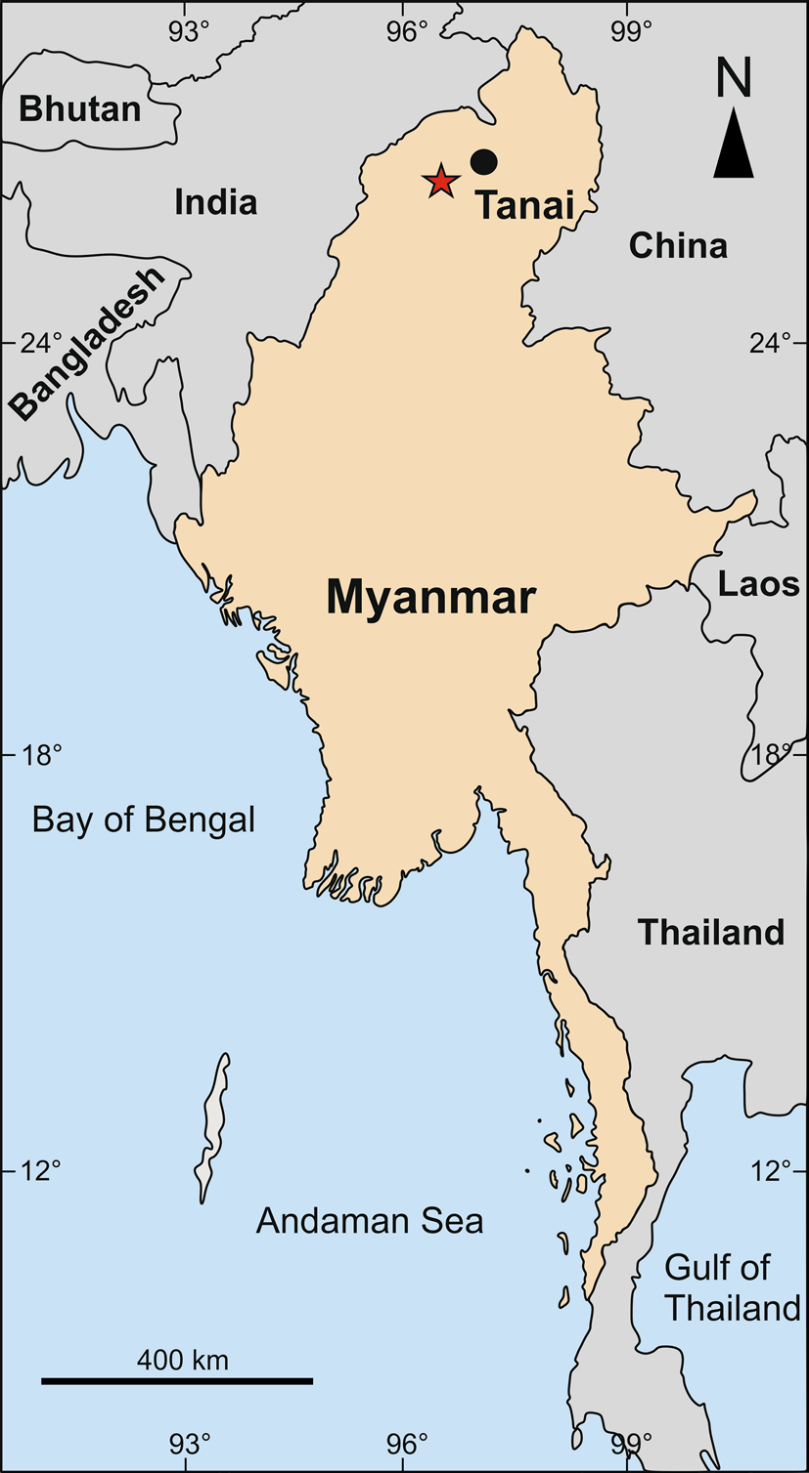
Map of Myanmar. The star designates the location of the Kachin amber mines, in Kachin State, near Tanai (Danai). The amber is early Cenomanian (~ 99 Ma, Late Cretaceous) in age.

The Cretaceous was a time of substantial environmental and biotic change. Since the first definitive record of angiosperms in the Early Cretaceous during the Valanginian–early Hauterivian at ca. 135 Ma^36,37^—although some authors defend an earlier origin^38^, see^39^—flowering plants underwent an increasingly rapid diversification and came to dominate most habitats by the end of the Cenomanian at ca. 94 Ma during the Late Cretaceous, replacing most of the previously existing, gymnosperm-dominated flora^36,40,41^. For plant-associated insects, the rapid replacement in existing niches of taxa co-associated with gymnosperms by new taxa co-associating with angiosperms (Fig. 3 in^4^) generally was made possible by modifying pre-existing structural adaptations with gymnosperms (direct or indirectly), and by evolution of novel structures connected with speciation events involving angiosperms^42^. Nevertheless, family-level insect diversity seems not to have increased during the global Cretaceous gymnosperm-to-angiosperm transition^12,14,18,43^, at least in most of the groups^20^. Some data show a slight decline of family-level insect diversity into the Late Cretaceous^13,19^. Three processes separately or jointly account for this trend. First is the overall balance of the family-level extinction rate with origination rate, or possibly a slight excess of the extinction rate over the origination rate, resulting in quickened lineage turnover. A second process is the absence of extinction and origination with the prolongation of lineage durations across multiple geological stages. A third process would be for all evolutionary turnover, including extinction and origination, to occur below the rank of the family, involving species, genera and tribes. These macroevolutionary processes provide multiple ways by which insect lineages would accommodate to an angiosperm-dominated world.

Clarke et al.^44^ described a significant number of new species in the newly erected fossil weevil family Mesophyletidae from Kachin amber. This speciose family exhibited specialized characters for herbivory and possibly pollination, whose hosts the authors concluded were most likely early magnoliid-grade angiosperms^44^. If Mesophyletidae weevils were adapted to angiosperms, a clade that became highly successful evolutionarily, it is intriguing to ascertain how this diverse family of beetles from the Cretaceous—consisting of two subfamilies, 25 genera and 58 described species—became extinct, and why they were so abundantly found in gymnosperm resin, after the demonstrated relationship that trapped organisms had with the resin producing trees^45^. Perhaps the proposed association of this clade of fossil weevils with angiosperms should be re-evaluated^46^. A more likely explanation for the extinction of this clade is that their demise followed the evolutionary extinction of their gymnosperm hosts. Mesophyletidae may be one of the more remarkable examples of the demise of a diverse clade of insects co-associated with gymnosperm hosts that was unable to make the transition onto angiosperm hosts. Such an event would parallel the evolutionary fate of most of the approximately 50 taxa of mid-Mesozoic, long-proboscid pollinator lineages of Mecoptera, Neuroptera, and Diptera with direct and indirect evidence for associations with gymnosperms^4,5,47–50^. Other families described from Cretaceous ambers, including Mysteriomorphidae, currently represent speciose poor lineages whose life-habits also remain unknown, but nevertheless share sporadic appearances in the amber record.

The extinction of these families, including Mysteriomorphidae, most likely took Mode 1 of Peris et al.^4^, consisting of species with previous gymnosperm associations that did not survive the transition from a gymnosperm-dominated to an angiosperm-dominated flora. This association does not necessarily imply pollination of host plants. Indeed, most beetle families found in Cretaceous amber have a saproxylic lifestyle^7,46^, as perhaps did Mysteriomorphidae. Because the very source of the Cretaceous amber, many families of beetles possessed an evident gymnosperm saproxylic lifestyle, which became disrupted by the evolutionary and ecological success of angiosperms. The exceptional diversity of families during this time and their absence in the subsequent fossil record or presence in the modern biota supports such an explanation. Many insect families from Cretaceous ambers with extant representatives^8,46^ followed Mode 2, consisting of insects with ancestral gymnosperm host associations that mostly did not make the transition to angiosperms, some of which survived in highly decreased diversity to the present day; or alternatively, followed Mode 3 that represents a successful complete transition from a gymnosperm to angiosperm dominated flora to the present data^4,5^. Mode 4, in which the clade solely evolved with angiosperms and had no gymnosperm host associations in its history^4^, is a second possibility for lineages described during this interval. Nevertheless, the apparently rapid extinction of lineages such as the diverse Mesophyletidae or their absence in the Cenozoic or modern record strongly mitigates against such an explanation. Although there are plant-associated beetle clades originating during this time interval of early angiosperm diversification that might have co-diversified and co-associated with angiosperms^10,11,15^, some of these examples are debatable^2,9,17^. Collectively, these data indicate that the evolution of life habits directly and indirectly associated with angiosperm diversification probably resulted in major impact on beetle diversity during this formative interval, including the demise of Mysteriomorphidae and other beetle families, forcing their evolution or resulting in extinction.

## Conclusions

The fossil family Mysteriomorphidae received its name because authors could not assess its position within Elateriformia based on originally available morphological characters. The newly examined material from the same deposit and the CT-scan of one of the specimens have allowed re-description of the family and its final placement in Elateroidea, near Elateridae. Mysteriomorphidae is one of the six families that uniquely occurred in Cretaceous ambers during an interval of time coincident with angiosperm diversification. Although plant-associated insects, such as Coleoptera that is the target group in our work, had occupied niches involving various types of herbivory and pollination of gymnosperms for tens of millions of years earlier, the rise of angiosperms frequently allowed these niches to be rapidly occupied by new, ecologically analogous groups that displaced pre-existing, gymnosperm-associated groups, leading to their extinction. This extinction of pre-existing, directly or indirectly gymnosperm associated clades likely was a continual process whereby more newly evolved plant-associated clades replaced existing ones, the result of which was a leveling of overall plant-associated insect diversity that lasted for most of the Cretaceous Period.

## Material and methods

The material for this study comes from the Hukawng Valley, located near Noije Bum Mountain, 20 km southwest of Tanai (or Danai), in Kachin Province of northern Myanmar (Fig. 3)^51^, hereafter designated as “Kachin amber”. The most recently established age for Kachin amber is early Cenomanian, 98.79 ± 0.62 Ma^28^. However, some authors have proposed an earlier age such as a late Albian–early Cenomanian boundary date^52^, creating confusion in some reports which cited a substantially earlier age for Kachin amber without geochronometric support.

Four separate samples of amber containing four specimens of Mysteriomorphidae, were studied: NIGP173648, NIGP173649, NIGP173650, and NIGP173651. All specimens are deposited at the Nanjing Institute of Geology and Palaeontology, at Nanjing, China, and were examined under a Leica MZ95 stereomicroscope and a Leica DME compound microscope. Detailed photomicrographs were created and merged using a Keyence VHX1000 digital microscope under incident light. The specimen NIGP173648 has been figured by Peris^8^. The specimen NIGP173651 was scanned using a phoenix x-ray v tomex s 180 micro-computed-tomography scanner (GE Measurement & Control, Wuntsdorf, Germany), housed at the Institute of Geosciences at the University of Bonn. The data set has a resolution of 4.24 µm and the scan was carried out at 80 kV and 160 µA. Three frames per projection were acquired by a timing of 500 ms for a total of 1000 projections. The CT data were processed using the software VGSTUDIO MAX 3.2 (Volume Graphics, Heidelberg, Germany) and Avizo Lite 2019.4 (Thermo Scientific, Schwerte, Germany) to visualize the entire specimen that also complemented structures identified using optical microscopes. Final images were created by using CorelDraw 2018. All relevant structures were measured from digitized images. The re-examination of the type material, deposited in the amber collection of the Center for Natural History (CenNak), University of Hamburg, Germany, was unnecessary because Dr. Alekseev, the first author of the original description, shared with us all specimen illustrations in his possession. For Elateriformia, we follow the classification by Kundrata et al.^25^, with subsequent changes by Bocak et al.^30^, Kusy et al.^31,32^, Kundrata et al.^53^, and Rosa et al.^26^.

## Data Availability

All the samples studied in this work are available at the public collection of the Nanjing Institute of Geology and Palaeontology, at Nanjing, China.
